# Hypoxia-inducible factor 1α inhibitor reduces hippocampal neuronal ferroptosis

**DOI:** 10.4103/NRR.NRR-D-24-01007

**Published:** 2025-02-24

**Authors:** Zhen Liang, Qi Guo, Zhaoshi Zheng, Yingyue Lou, Xiaojuan Zhu, Songyan Liu

**Affiliations:** 1 Department of Neurology, China-Japan Union Hospital of Jilin University, Changchun, Jilin Province, China; 2 Department of Rehabilitation, The Second Hospital of Jilin University, Changchun, Jilin Province, China; 3 Key Laboratory of Molecular Epigenetics, Ministry of Education and Institute of Cytology and Genetics, Northeast Normal University, Changchun, Jilin Province, China

**Keywords:** epilepsy, ferroptosis, heme oxygenase-1, hippocampus, hypoxia-inducible factor 1α, mitochondrial ultrastructure, oxidative stress, PX-478, reactive oxygen species, seizure behavior

## Abstract

Epilepsy is a prevalent neurological disorder in which hippocampal neuronal damage, particularly ferroptosis, plays a critical role. Previous studies have shown that hypoxia-inducible factor 1α is considered an important regulator of cellular stress responses and has been confirmed to play a critical role in the occurrence of various diseases. However, the mechanisms by which hypoxia-inducible factor 1α is related to epilepsy and neuronal ferroptosis remain unclear. In this study, we used a pentylentetrazole-induced chronic epilepsy mouse model and treated the mice with intraperitoneal administration of PX-478, a hypoxia-inducible factor-1α inhibitor. Our results showed that PX-478 significantly prolonged the latency of epilepsy, reduced seizure severity, and shortened seizure duration. PX-478 also alleviated neuronal damage in the hippocampal CA1 and CA2 regions, reduced levels of reactive oxygen species and malondialdehyde, and increased levels of superoxide dismutase, catalase, and glutathione peroxidase. Transmission electron microscopy showed that PX-478 treatment reduced mitochondrial damage in the hippocampal neurons of epileptic mice, and significantly improved mitochondrial length and area. Additionally, PX-478 preferentially reduced Fe^2+^ levels and the expression of cyclooxygenase-2, ferritin heavy chain 1 and transferrin in the hippocampus of epileptic mice. It also inhibited the activity of the hypoxia-inducible factor 1α/heme oxygenase-1 pathway. In summary, these findings suggest that PX-478 has the potential to treat epilepsy by inhibiting the hypoxia-inducible factor 1α/heme oxygenase-1 pathway, alleviating oxidative stress, and reducing ferroptosis in hippocampal neurons.

## Graphical abstract

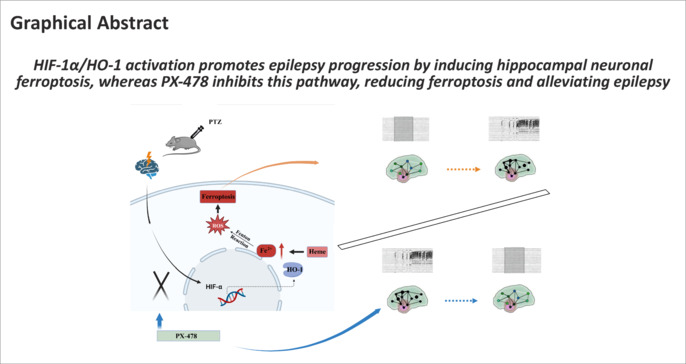

## Introduction

Epilepsy is a neurological disorder characterized by highly synchronized abnormal discharges of brain neurons, resulting in spontaneous, recurrent, and transient dysfunction of the central nervous system (Scheffer et al., 2017; Jiao et al., 2025). This chronic condition leads to neuronal loss and abnormal distribution in cortical lesion areas of the brain, with hippocampal sclerosis being a pivotal aspect of its pathological process (Thom, 2014). Oxidative stress plays a crucial role in the onset, development, and manifestation of epilepsy, as evidenced by numerous animal experiments and clinical studies (Mao et al., 2019; Ismy et al., 2024; Li et al., 2024), and is a primary cause of hippocampal neuron death (Sun et al., 2022). Increased seizure frequency correlates positively with oxidative stress markers. In genetically susceptible rats, endogenous antioxidant enzyme activity decreases, accompanied by elevated levels of lipid peroxidation and protein oxidation markers. Key biomarkers of DNA oxidative damage, such as 8-hydroxy-2ʹ-deoxyguanosine, are significantly elevated in both acute and chronic epilepsy models (Patel, 2004; Lin et al., 2010). Oxidative stress is a primary cause of neuronal injury in epilepsy patients, leading to the accumulation of reactive oxygen species (ROS) and reactive nitrogen species and widespread intracellular molecular damage, including lipid peroxidation, DNA damage, protein oxidation, and cellular dysfunction, and ultimately resulting in cell death (Méndez-Armenta et al., 2014; Vishnoi et al., 2016). Additionally, oxidative stress in epilepsy patients triggers ferroptosis, which plays a central role in epilepsy onset and neuronal demise (Petrillo et al., 2021; Li et al., 2024). Globally, there are over 70 million epilepsy patients, with nearly 80% residing in low and middle-income countries (Thijs et al., 2019). Although antiepileptic drugs remain the most effective treatment for epilepsy, more than 30% of patients progress to drug-resistant epilepsy (Golyala and Kwan, 2017). Current therapeutic approaches targeting oxidative damage and ferroptosis in epilepsy patients have shown limited clinical efficacy. Therefore, a deeper exploration of potential therapeutic targets for epilepsy, particularly drug-resistant epilepsy, holds critical importance in improving clinical outcomes.

Hypoxia-inducible factor-1α (HIF-1α) is an essential transcription factor within the HIF-1 family. HIF-1α serves as the primary functional component, playing a critical role in activating the transcription of more than 60 genes, including heme oxygenase-1 (HO-1), especially in low-oxygen environments. This mechanism enables cells to adjust to hypoxic conditions. HIF-1α’s stability and function are modulated by several factors, including blood oxygen levels, intracellular iron concentration, and the activation of various signaling pathways (Otterbein et al., 2003; Belaidi et al., 2016). During epileptic seizures, intense neuronal electrical activity may increase energy consumption and oxygen demand, potentially leading to localized hypoxia and subsequent activation of HIF-1α. Seizures may also exacerbate inflammation and disrupt neurovascular coupling, affecting cerebral blood flow and oxygen delivery, which could further activate HIF-1α. Moreover, seizures may elevate oxidative stress levels, potentially triggering HIF activation (Bateman et al., 2008; Moseley et al., 2010; Feast et al., 2012). Activation of HIF-1α during seizures may represent a protective response to hypoxic stressors induced by changes in blood flow or energy consumption, facilitating angiogenesis and erythropoiesis to prevent or alleviate hypoxic damage. Recent research has highlighted that, in addition to facilitating hypoxic adaptation, HIF-1α triggers ferroptosis in specific disease conditions, including malignant mesothelioma and diabetic nephropathy (Feng et al., 2021; Tang et al., 2021). HO-1 is a stress-inducible enzyme and the sole pathway for cellular heme degradation, metabolizing heme into biliverdin, carbon monoxide, and Fe^2+^ (which induces a ferritin increase) (Otterbein et al., 2003; Wang et al., 2024). Therefore, HO-1, known as the “guardian gene,” counterbalances the increased heme pool, further influencing intracellular iron levels (Lawen and Lane, 2013). Increased HO-1 levels lead to ferrous iron overload, enhancing the Fenton reaction, in which normally inert O_2_^–^ and H_2_O_2_ are transformed into highly reactive OH, causing lipid peroxidation and ultimately triggering ferroptosis (Hassannia et al., 2018). Studies have implicated the HIF-1α/HO-1 pathway in mediating ferroptosis in diabetic nephropathy and infertility (Feng et al., 2021; Wu et al., 2022). Furthermore, this pathway was shown to exacerbate lung injury induced by polystyrene nanoplastic-induced ferroptosis in pulmonary tissue cells (Wu et al., 2024). Following epileptic seizures, the brain undergoes a sequence of molecular, biochemical, metabolic, and structural changes, constituting a chronic process of brain injury that ultimately results in neuronal damage, ion channel dysfunction, mossy fiber sprouting, gliosis, altered synaptic plasticity, and inflammatory responses, with hippocampal neuronal damage and death being particularly pronounced (Gan et al., 2015). Hippocampal neuronal ferroptosis is a critical mechanism driving neuronal injury and seizure onset. Our prior research indicated that the increase of HIF-1α/HO-1 expression significantly influenced ferroptosis in hippocampal neurons after epilepsy onset, and thereby played a pivotal role in seizure development (Liang et al., 2023a).

PX-478 (S-2-amino-3-[4′-N,N,-bis(2-chloroethyl)amino]phenyl propionic acid N-oxide dihydrochloride) is an inhibitor of constitutive and hypoxia-induced levels of HIF-1α, and thereby reduces HIF-1 activity. PX-478 inhibits HIF-1α ubiquitination, leading to the degradation of polyubiquitinated HIF-1α, which in turn lowers HIF-1α mRNA expression and affects HIF-1α translation. It can decrease HIF-1α protein expression and transcriptional activity under both normoxic and hypoxic conditions (Koh et al., 2008). Notably, unlike other HIF-1α inhibitors, PX-478 exhibits low oral toxicity and can penetrate the blood–brain barrier, making it more suitable for therapeutic use (Schito and Semenza, 2016)..

Our previous research showed significant HIF-1α activation during epileptic pathologies, which was closely associated with hippocampal neuronal ferroptosis (Liang et al., 2023a). This provides a theoretical basis for the potential application of PX-478 in epilepsy treatment. However, direct studies on PX-478 in epilepsy treatment remain limited, and further research is needed to clarify its specific mechanisms and efficacy. This study investigated the effects of PX-478 on the HIF-1α/HO-1 signaling pathway, oxidative stress, hippocampal neuronal ferroptosis, and seizure occurrence, and aimed to evaluate PX-478 as a potential antiepileptic drug.

## Methods

### Animals and study design

The protocols for animal experiments were approved by the Institutional Animal Care and Use Committee of Jilin University, China (approval No. KT 202306149) on June 21, 2023. All experiments were designed and reported according to the Animal Research: Reporting of *In Vivo* Experiments (ARRIVE) guidelines (Percie du Sert et al., 2020).

**Figure 1:**
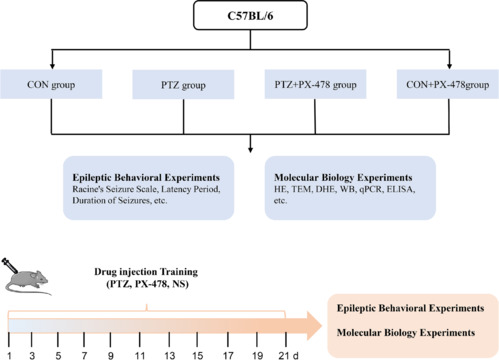
Experimental design. Each behavioral testing and molecular biology experiment was repeated at least three times. CON: Control; DHE: dihydroethidium; ELISA: enzyme linked immunosorbent assay; HE: hematoxylin-eosin; NS: normal saline; PTZ: pentylenetetrazol; PX-478: a hypoxia-inducible factor-1α inhibitor; qPCR: quantitative reverse transcription polymerase chain reaction; TEM: transmission electron microscopy; WB: western blot.

Eight-week-old male C57BL/6 mice, weighing 20.1 ± 2.3 g, were sourced from Yisi Laboratory Animal Technology Co., LTD. (Changchun, China; animal license No. SCXK2023-0002). The experimental animals were housed in a controlled environment with a temperature of 20–24°C, humidity of 40%–60%, and a 12-hour light/dark cycle, with free access to food and water to ensure their well-being. PTZ was used according to a previous study (Hu et al., 2023); a dosage of 35 mg/kg was administered by intraperitoneal injection. Using a random number table, the mice were randomly assigned into four groups (*n* = 16 per group) (**Figure 1**): (1) CON group: received two intraperitoneal injections of normal saline (0.2 mL), 30 minutes apart, every other day, for a total of 11 times; (2) PTZ group: received an intraperitoneal injection of normal saline (0.2 mL) followed by 35 mg/kg PTZ (Sigma-Aldrich, St. Louis, MO, USA, P6500) 30 minutes later, every other day, for a total of 11 times; (3) PTZ + PX-478 group: received an intraperitoneal injection of 5 mg/kg PX-478 (MedChemExpress, Monmouth Junction, NJ, USA, HY-10231) followed by 35 mg/kg PTZ 30 minutes later, every other day, for a total of 11 times; (4) CON + PX-478 group: received an intraperitoneal injection of 5 mg/kg PX-478 followed by normal saline (0.2 mL) 30 minutes later, every other day, for a total of 11 times.

### Epileptic seizure behavior testing

Seizure activity was recorded by video for 30 minutes after each injection. Seizure level was assessed using the modified Racine scale. In brief, the grading criteria are as follows: stage 0, no response; stage 1, ear and facial twitching; stage 2, convulsive twitching through the body; stage 3, myoclonic jerks and rearing; stage 4, wild running and jumping; stage 5, generalized tonic-clonic seizures; and stage 6, death (Schröder et al., 1993). Kindling was deemed successful if stage 4–5 seizures were observed in three consecutive PTZ injections. For fully kindled mice, both the kindling latency and the latency and duration of seizures exceeding stage 4 after the final injection were recorded.

### Hematoxylin and eosin staining

Mice were anesthetized using a continuous gas mixture of 2% isoflurane (RWD, Shenzhen, China; anesthetic consumption rate: 3.6 mL/h). After cardiac perfusion, the brains were carefully extracted and post-fixed in 4% paraformaldehyde overnight at 4°C to ensure thorough fixation. Following fixation, the brains were transferred to a 30% sucrose solution for cryoprotection and were left in the sucrose solution until they sank to the bottom, which typically took 24–48 hours. Once cryoprotected, the brains were embedded in paraffin wax and then sectioned into 6-µm-thick slices using a microtome. These sections were mounted on glass slides for hematoxylin and eosin (HE) staining. The staining process began with deparaffinization of the sections in xylene, followed by rehydration through a series of graded ethanol solutions and finally rinsing in distilled water. The sections were then stained with hematoxylin for 5–10 minutes to visualize the cell nuclei, followed by rinsing under running tap water for a few minutes. Differentiation was performed using 1% acid alcohol, and the sections were then returned to a bluing solution, typically 0.5%–1% ammonia water, to enhance the hematoxylin staining (Beyotime, Shanghai, China, C0105S), followed by a final rinse in running tap water. Next, the sections were stained with eosin for 2–5 minutes to visualize the cytoplasm and other tissue structures. After eosin staining, the sections were dehydrated through graded alcohols, cleared in xylene, and finally mounted with a neutral resinous medium to preserve the sections. Images of the stained sections were captured using an Olympus (Tokyo, Japan, FSX100) microscope, allowing for detailed histological examination of the brain tissue.

### Transmission electron microscopy

Mice were perfused transcardially with heparinized saline, followed by 2.5% glutaraldehyde and 1% paraformaldehyde in 0.1 M phosphate buffer (pH 7.4). The hippocampus was then dissected and prepared for transmission electron microscopy (TEM) as previously described (Ma et al., 2019). Briefly, the hippocampal tissue was diced into small pieces (approximately 1 mm^3^) to ensure optimal fixation. These tissue samples were then placed in fresh 2.5% glutaraldehyde in 0.1 M phosphate buffer and fixed at 4°C for several hours to preserve cellular components. After fixation, the tissue samples were sectioned into 70-µm-thick slices. Following osmication, the sections were thoroughly dehydrated through a graded series of ethanol solutions, from 50% to 100%, and then transitioned through propylene oxide. Next, the dehydrated sections were embedded flat in EMbed 812 resin (EMS, Hatfield, PA, USA, A57631), which was polymerized at 60°C for 48 hours to form hardened blocks suitable for ultrathin sectioning. Ultrathin sections, approximately 60 nm in thickness, were cut from these resin blocks using an ultramicrotome equipped with a diamond knife. These sections were carefully collected on Formvar-coated single-slot copper grids. The sections were then stained with uranyl acetate, typically for 20 minutes, to enhance contrast by binding to nucleic acids and proteins, followed by a secondary staining with lead citrate for 5–10 minutes to further enhance the contrast of cellular structures. The stained sections were examined using a Hitachi (Tokyo, Japan, HT-7800) transmission electron microscope, allowing for high-resolution visualization of the hippocampal ultrastructure.

### Reverse transcription quantitative polymerase chain reaction

The assays were conducted as reported previously (Yu et al., 2019; Liang et al., 2023b). Briefly, hippocampal tissue was homogenized in TRIzol reagent (Invitrogen, Carlsbad, CA, USA, Cat# 257401) according to the manufacturer’s protocol, and the RNA was extracted by phase separation using chloroform, followed by precipitation with isopropanol. The RNA pellet was then washed with 75% ethanol, air-dried, and resuspended in RNase-free water. The concentration and purity of the RNA were assessed using a NanoDrop spectrophotometer (Invitrogen). Then, 1 µg of total RNA was reverse transcribed into complementary DNA. The TransScript® Green One-Step Reverse transcription quantitative polymerase chain reaction (RT-qPCR) SuperMix (TransGen, Beijing, China, Code# AQ211-01) includes both genomic DNA removal and reverse transcription components, allowing for efficient complementary DNA synthesis in a single reaction. The reaction mixture was prepared according to the manufacturer’s instructions and incubated at 42°C for 15 minutes for reverse transcription, followed by inactivation of the reverse transcriptase at 85°C for 5 seconds. RT-qPCR was then carried out using SYBR Premix Ex Taq^TM^ II (Takara, Otsu, Japan, Cat# RR820A) on an ABI StepOnePlus Real-Time PCR System (Applied Biosystems, Waltham, MA, USA). Each reaction was performed in 20 µL volume containing 10 µL of SYBR Green master mix, 0.4 µM of each forward and reverse primer, 2 µL of complementary DNA template, and nuclease-free water. The PCR conditions were as follows: initial denaturation at 95°C for 30 seconds, followed by 40 cycles of 95°C for 5 seconds and 60°C for 30 seconds. Each sample was analyzed in triplicate to ensure the accuracy of the results. The expression levels of the target genes were normalized to the housekeeping gene β-actin using the 2^–∆∆CT^ method (Liang et al., 2023a), which allows for relative quantification of gene expression. After the amplification cycles, melting curve analysis was automatically performed to verify the specificity of the PCR products, ensuring that only a single peak was present for each reaction (**Table 1**).

**Table 1:** Primer sequences information

Gene	Primer sequence
Mouse *β-actin*	F: 5'-GGT GAA GGT CGG TGT GAA CG-3' R: 5'-CTC GCT CCT GGA AGA TGG TG-3'
Mouse *HIF-1α*	F: 5'-GAA TGA AGT GCA CCC TAA CAA G-3' R: 5'-GAG GAA TGG GTT CAC AAA TCA G-3'
Mouse *HO-1*	F: 5'-CCG CTA CCT GGG TGA CCT CTC-3' R: 5'-GAC GAA GTG ACG CCA TCT GTG AG-3'
Mouse *GPX4*	F: 5'-CAT GCC CGA TAT GCT GAG TGT GG-3' R: 5'-TAG CAC GGC AGG TCC TTC TCT ATC-3'
Mouse *FTH1*	F: 5'-TGC CAT CAA CCG CCA GAT CAA C-3' R: 5'-ATT CAG CCC GCT CTC CCA GTC-3'
Mouse *Transferrin*	F: 5'-GGA CGC CAT GAC TTT GGA TG-3' R: 5'-GCC ATG ACA GGC ACT AGA CC-3'
Mouse *PTGS2*	F: 5'-TTC CAA TCC ATG TCA AAA CCG T-3' R: 5'-AGT CCG GGT ACA GTC ACA CTT-3'

F: Forward; FTH1: ferritin heavy chain 1; GPX4: glutathione peroxidase 4; HIF-1α: hypoxia-inducible factor 1α; HO-1: heme oxygenase-1; PTGS2: prostaglandin synthase 2; R: reverse.

### Western blotting

Mouse hippocampal tissue was lysed using a modified radioimmunoprecipitation assay buffer (50 mM Tris-HCl, pH 7.4, 150 mM sodium chloride, 1% NP-40, 0.25% sodium deoxycholate, and proteinase inhibitors). Protein concentrations were determined with a BCA protein assay kit (P0012, Beyotime). Western blotting was performed as previously described (Yu et al., 2019). Proteins were separated by sodium dodecyl sulfate-polyacrylamide gel electrophoresis and transferred onto polyvinylidene fluoride membranes (Millipore, Darmstadt, Germany). Membranes were incubated overnight at 4°C with specific primary antibodies, followed by horseradish peroxidase (HRP)-conjugated secondary antibodies at 24–26°C for 1 hour. Chemiluminescent signals were visualized using ECL Prime Western Blot Detection reagent (GE Healthcare, Chicago, IL, USA) and detected with a Tanon-5200 system (Tiangen (Beijing) Biotech Co., Ltd.). The optical densities (ODs) of bands were quantified using ImageJ software (v1.53t, National Institutes of Health, Bethesda, MD, USA; Schneider et al., 2012) and normalized to β-actin levels. The following antibodies were used: rabbit anti-HO-1 (1:10,000, Abcam, Cat# AB68477, RRID: AB_11156457); rabbit anti-HIF-1α (1:1000, Abmart, Shanghai, China, Cat# P50517R1, RRID: AB_ 3665517), rabbit anti-glutathione peroxidase 4 (GPX4; 1:1000, Abmart, Cat# T56959F, RRID: AB_2936461), rabbit anti-transferrin (1:1000, Abmart, Cat# T40111F, RRID: AB_2936323), rabbit anti-ferritin (1:1000, Abmart, Cat# T55648F, RRID: AB_2936974), rabbit anti-cyclooxygenase-2 (COX2; 1:1000, Abmart, Cat# T58852F, RRID: AB_2936469), mouse anti-β-actin (1:5000, Sigma-Aldrich, Cat# A1978, RRID: AB_476692), donkey anti-mouse IgG (1:1000, Invitrogen, Cat# A10036, RRID: AB_2534012) and donkey anti-rabbit IgG (1:1000, Invitrogen, Cat# A10040, RRID: AB_2534016).

### Enzyme-linked immunosorbent assay

Ferritin and transferrin protein levels in mouse hippocampus lysates were quantified using specific enzyme-linked immunosorbent assay (ELISA) kits (ferritin: Cat# LE-M2088, transferrin: Cat# LE-M1662, Lai Er Bio-Tech, Hefei, Anhui, China), following the protocols provided by the manufacturer. Hippocampal tissues were homogenized in ice-cold lysis buffer containing protease inhibitors to prevent protein degradation. The homogenates were then centrifuged at 12,000 × *g* for 15 minutes at 4°C to remove debris, and the supernatants were collected for protein quantification. The total protein concentration in each lysate was determined using the Bradford method, which involves adding Bradford reagent to the samples and measuring the absorbance at 595 nm to calculate the protein concentration based on a standard curve. For the ELISA, aliquots of the prepared lysates were loaded into the wells of a 96-well microplate pre-coated with antibodies specific to ferritin or transferrin. The samples were incubated at 37°C in a water bath or an incubator for 60 minutes to allow the target proteins to bind to the antibodies. After incubation, the wells were washed multiple times with the provided wash buffer to remove unbound proteins. Next, a biotinylated detection antibody specific to either ferritin or transferrin was added to each well, followed by incubation. This was followed by the addition of a streptavidin-HRP conjugate, which binds to the biotinylated antibody. The plates were then washed to remove any unbound enzyme conjugate. The bound enzyme was detected by adding the substrate solution, which reacts with the HRP to produce a color change. The reaction was stopped by adding the stop solution, and the OD of each well was measured at 450 nm using a Milton Roy Spectronic 3000 Array spectrophotometer (Milton Roy, Shanghai, China). The concentration of ferritin and transferrin in each sample was determined by comparing the OD values to a standard curve generated from known concentrations of the target proteins.

### Ferrous iron (Fe^2+^) assay

The relative concentration of Fe^2+^ in mouse hippocampal tissue lysates was measured using an Iron Assay Kit (Cat# MAK025, Sigma-Aldrich) according to the manufacturer’s instructions. Mouse hippocampal tissue was homogenized in ice-cold extraction buffer with protease inhibitors to prevent protein degradation. The homogenates were then centrifuged at 12,000 × *g* for 15 minutes at 4°C, and the supernatant containing the tissue lysate was collected. Total protein content in each sample was measured using the Bradford method. For the Fe^2+^ assay, aliquots of the lysates were mixed with the assay reagent, and the reaction was allowed to proceed at room temperature (23 ± 2°C) as specified by the kit protocol. The OD of the samples was then recorded at 593 nm using a Milton Roy Spectronic 3000 Array spectrophotometer. The Fe^2+^ concentration in each sample was determined by comparing the OD values to a standard curve generated with known Fe^2+^ concentrations provided by the assay kit.

### Antioxidant enzyme activity assay

The activity of superoxide dismutase (SOD), catalase (CAT), and glutathione peroxidase (GPx) in mouse hippocampal tissue was assessed using commercial test kits (S0101S, S0051, and S0056, Beyotime) according to the manufacturer’s protocols. Absorbance was measured at 450 nm for SOD, 340 nm for GPx, and 520 nm for CAT using a Milton Roy Spectronic 3000 Array spectrophotometer. Protein content in each sample was determined using the Bradford method.

### Reactive oxygen species staining

For ROS staining, mice were anesthetized using a continuous gas mixture of 2% isoflurane (RWD, Shenzhen, China; anesthetic consumption rate: 3.6 mL/h), and their brains were immediately dissected and snap-frozen in liquid nitrogen to preserve the tissue. The frozen brains were stored at –80°C until further processing. To prepare the tissue for sectioning, the brain samples were embedded in optimal cutting temperature compound (Cat# G6059, Wuhan Servicebio Technology CO., LTD, Wuhan, China) and allowed to solidify at –20°C. A cryostat microtome (CRYOSTAR NX50; Thermo Fisher Technology, Shanghai, China) was used to cut 8-µm-thick brain sections from the frozen blocks. The sections were mounted on glass slides and briefly thawed at room temperature. To remove any cryoprotectant and to rehydrate the tissue, the slides were washed with distilled water. The ROS staining was performed by incubating the sections in dihydroethidium (DHE) staining solution (Cat# D7008, Sigma-Aldrich) for 30 minutes at 37°C in a humidified chamber to ensure optimal staining conditions. Following DHE incubation, the sections were stained with nucleus staining solution (Cat# G1012, Wuhan Servicebio Technology CO., LTD) for 10 minutes to label the cell nuclei. After staining, the slides were washed with phosphate-buffered saline to remove excess dye. The ROS-stained sections were then examined using a LSM 780 confocal microscope (Zeiss, Oberkochen, Germany) with ZEN 2012 software. During image acquisition, adjustments to brightness, contrast, and color balance were made post-imaging to enhance visualization of the ROS fluorescence. For quantification of ROS fluorescence intensity, the images were analyzed using ImageJ software. The fluorescence intensity in the hippocampal regions was measured, and data were normalized to account for variations in background staining and section thickness.

### Lipid peroxidation assay

Malondialdehyde (MDA) concentration in mouse hippocampal lysates was measured using a lipid peroxidation MDA assay kit (S0131S, Beyotime) according to the manufacturer’s instructions. Optical density values were recorded at 532 nm using a Milton Roy Spectronic 3000 Array spectrophotometer. Protein content in each sample was determined using the Bradford method.

### Statistical analysis

Statistical analysis was conducted using IBM SPSS Statistics for Windows (Version 25.0, IBM Corp., Armonk, NY, USA). Before performing any analyses, the normal distribution of the data was confirmed using the Shapiro–Wilk test. For comparing two groups, Student’s *t*-test was used. For comparing three or more groups, one-way analysis of variance was used. *Post hoc* Tukey’s honest significant difference test was used when necessary to identify specific group differences. Data are expressed as the mean ± standard error of the mean. Statistical significance was defined as *P* ≤ 0.05. Graphical representations of the data were created using GraphPad Prism 8.0.1 (GraphPad Software, Boston, MA, USA, www.graphpad.com).

## Results

### PX-478 inhibits epileptic seizure behaviors in mice

**Figure 2:**
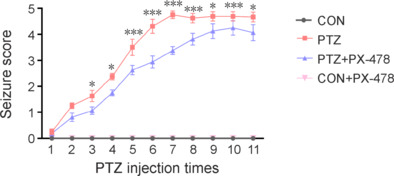
Seizure scores after each injection of PTZ in mice. Adult male C57BL/6 mice were assigned to four experimental groups: the CON group received saline only; the PTZ group received saline followed by 35 mg/kg of PTZ; the PTZ + PX-478 group received 5 mg/kg of PX-478 before PTZ administration; and the CON + PX-478 group received PX-478 followed by normal saline. Data are expressed as mean ± SEM (*n* = 16). *P < 0.05, ***P < 0.001, PTZ *vs*. PTZ + PX-478 (Student’s *t*-test). CON: Control; PTZ: pentylenetetrazol; PX-478: a hypoxia-inducible factor-1α inhibitor.

We successfully established a PTZ-kindled epilepsy mouse model using previously established methods (Liang et al., 2023a). The study design is depicted schematically in **Figure 1**. Compared with the PTZ group, the PTZ + PX-478 group showed a longer kindling latency and increased latency to seizure after the final injection. Additionally, the seizure scores, full kindling success rates, and seizure duration after the final injection were significantly reduced in the PTZ + PX-478 group (**Table 2** and **Figure 2**).

**Table 2:** Behavioral results of each group

Group	n	Percentage of kindled	Latency time of kindled (d)	Last seizure scale ≥ 4	
				Latency (s)	Duration time (s)
CON	16	0	–	–	–
PTZ	16	75.0	16.00±1.21	206.00±10.92	62.15±3.39
PTZ+PX-478	16	56.3	19.11±1.05	215.56±9.86	48.56±4.72
CON+PX-478	16	0	–	–	–

Data are presented as mean ± SEM. CON: Control; PTZ: pentylenetetrazol; PX-478: a hypoxia-inducible factor-1α inhibitor.

### PX-478 alleviates neuronal damage in hippocampal CA1, CA3, and dentate gyrus regions of epilepsy model mice

**Figure 3:**
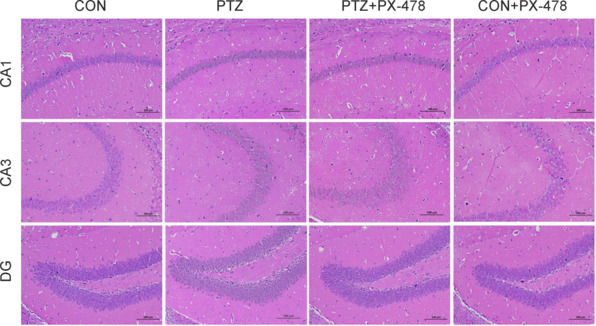
Effect of PX-478 on neuronal damage in hippocampal CA1, CA3, and DG regions of epilepsy model mice. In the CON and CON + PX-478 groups, neurons in the hippocampal CA1, CA3, and DG regions were organized and intact. In the PTZ group, neurons in CA1 and CA3 showed disorganized arrangement, loss, and abnormal morphology. DG damage was less severe. The PTZ + PX-478 group exhibited reduced neuronal damage in CA1 and CA3 compared with that in the PTZ group, though some neuronal loss remained. Scale bars: 100 μm. CON: Control; DG: dentate gyrus; PTZ: pentylenetetrazol; PX-478: a hypoxia-inducible factor-1α inhibitor.

To observe neuronal damage in different the treatment groups, we performed HE staining on brain tissue sections. In the CON and CON + PX-478 groups, neurons in the CA1, CA3, and dentate gyrus (DG) regions of the hippocampus were neatly arranged with intact structures, and no substantial neuronal loss was observed. In the PTZ group, neurons in the CA1 and CA3 regions exhibited disorganized arrangement, partial neuronal loss, and abnormal morphology characterized by smaller irregular shapes with increased color intensity and nuclear condensation. Damage in the DG region was less pronounced. Compared with the PTZ group, the PTZ + PX-478 group exhibited noticeably reduced neuronal damage in the CA1 and CA3 regions of the hippocampus. Nevertheless, some neuronal death and loss were still evident when compared with the CON group (**Figure 3**).

### PX-478 decreases the expression of oxidative stress indicators in the hippocampus of epilepsy model mice

**Figure 4:**
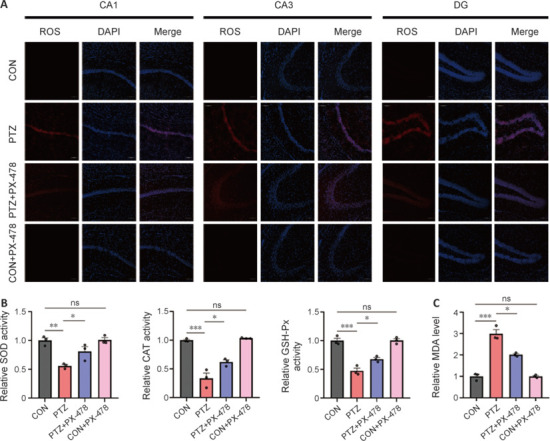
Effect of PX-478 on the expression of oxidative stress indicators in the hippocampus of epilepsy model mice. (A) ROS staining of CA1, CA3, and DG regions in hippocampus. Compared with the CON and CON + PX-478 groups, the PTZ group appeared to have higher ROS levels in all regions, which were reduced in the PTZ + PX-478 group. Scale bars: 100 μm. (B) Relative activity of antioxidant enzymes SOD, CAT, and GSH-Px in hippocampus. (C) Relative MDA content in hippocampus. The data, normalized to CON, are presented as mean ± SEM (*n* = 3). *P < 0.05, **P < 0.01, ***P < 0.001 (one-way analysis of variance followed by Tukey’s honestly significant difference test). CAT: Catalase; CON: control; DG: dentate gyrus; GSH-Px: glutathione peroxidase; MDA: malondialdehyde; ns: not significant; PTZ: pentylenetetrazol; PX-478: a hypoxia-inducible factor-1α inhibitor; ROS: reactive oxygen species; SOD: superoxide dismutase.

Excessive ROS accumulation from oxidative stress is a pivotal step that triggers ferroptosis (Li et al., 2020; Stockwell, 2022). We used DHE staining to assess ROS levels in the hippocampal CA1, CA3, and DG regions of each group. Compared with the CON and CON + PX-478 groups, the PTZ group appeared to have higher ROS levels in hippocampal CA1, CA3, and DG tissues, which were markedly reduced after PX-478 treatment (**Figure 4A**). ROS generation may be attributed to excessive Fe^2+^ triggering the Fenton reaction (Park and Chung, 2019). We next measured the activities of ROS-scavenging enzymes SOD, CAT, and GSH-Px in hippocampal tissue, and found that compared with the CON and CON + PX-478 groups, the PTZ group showed significantly decreased levels of these enzymes, which were significantly elevated after PX-478 treatment (**Figure 4B**). Excessive ROS accumulation attacks lipid membranes, leading to lipid peroxidation and subsequent ferroptosis. MDA, a natural byproduct of biological lipid oxidation, reflects the level of lipid oxidation and is widely used as an indicator of lipid peroxidation (Qin et al., 2021; Liu et al., 2022). Compared with the CON and CON + PX-478 groups, the PTZ group had significantly elevated MDA levels in hippocampal tissue, which were significantly reduced after PX-478 treatment (**Figure 4C**).

### PX-478 alleviates mitochondrial damage in hippocampal neurons of epilepsy model mice

**Figure 5:**
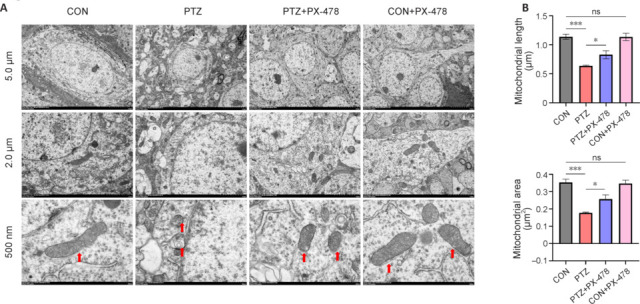
Effect of PX-478 on mitochondrial damage in hippocampal neurons of epilepsy model mice. (A) Transmission electron microscope images of hippocampus for each group with 5.0 μm, 2.0 μm, and 500 nm scales. In the CON and CON + PX-478 groups, the morphology and size of mitochondria in hippocampal neurons appeared normal. In the PTZ group, hippocampal neurons displayed smaller mitochondria with increased membrane density and reduced or absent cristae, whereas the PTZ + PX-478 group showed a notable reduction in mitochondrial damage. Arrows indicate mitochondria. (B) Quantification of mitochondrial length and area in the hippocampus. The data are presented as mean ± SEM (*n* = 5). *P < 0.05, ***P < 0.001 (one-way analysis of variance followed by Tukey’s honest significant difference test). CON: Control; ns: not significant; PTZ: pentylenetetrazol; PX-478: a hypoxia-inducible factor-1α inhibitor.

To elucidate the specific types of oxidative damage, we used TEM to observe hippocampal neurons in mice, aiming to assess subcellular structural changes across the different groups. TEM images indicated that the nuclear size of hippocampal neurons was normal across all groups, and no chromatin condensation was observed. However, compared with those in the CON and CON + PX-478 groups, hippocampal neurons in the PTZ group appeared to have smaller mitochondria with increased membrane density and reduced or absent cristae. Compared with the PTZ group, the PTZ + PX-478 group exhibited reduced mitochondrial damage. Nonetheless, some mitochondrial damage was still noticeable when compared with the CON and CON + PX-478 groups (**Figure 5A**). Statistical analysis indicated that mitochondrial length and area in hippocampal neurons of the PTZ + PX-478 group were increased compared with those in the PTZ group, and decreased compared with those in the CON and CON + PX-478 groups (**Figure 5B**).

### PX-478 decreases the expression of ferroptosis indicators in the hippocampus of epilepsy model mice

**Figure 6:**
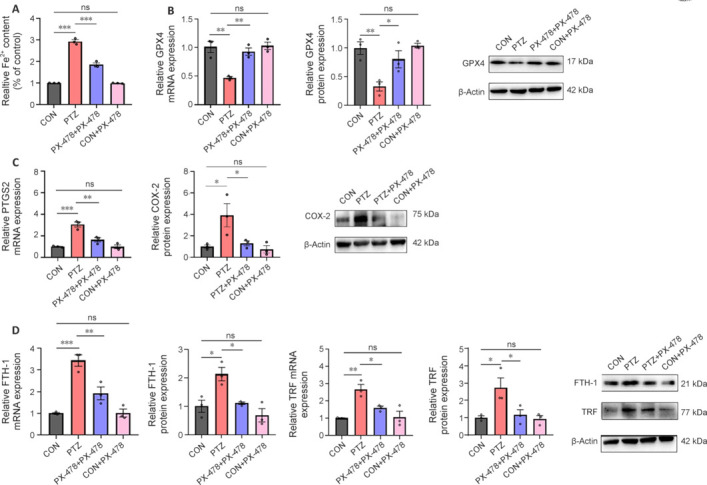
Effect of PX-478 on the expression of ferroptosis indicators in the hippocampus of epilepsy model mice. (A) Relative Fe^2+^ accumulation in the hippocampus. (B–D) RT-qPCR and western blot results for GPX4 (B), PTGS2/COX-2 (C), and FTH-1 and TRF (D) in the hippocampus. The data are presented as mean ± SEM (*n* = 3). *P < 0.05, **P < 0.01, ***P < 0.001 (one-way analysis of variance followed by Tukey’s honest significant difference test). CON: Control; COX-2: cyclooxygenase 2; FTH-1: ferritin heavy chain 1; GPX4: glutathione peroxidase 4; ns: not significant; PTGS2: prostaglandin synthase 2; PTZ: pentylenetetrazol; PX-478: a hypoxia-inducible factor-1α inhibitor; RT-qPCR: reverse transcription quantitative polymerase chain reaction; TRF: transferrin.

Given that the mitochondrial morphology observed with TEM was consistent with characteristic mitochondrial changes during ferroptosis (Chen et al., 2021), we subsequently measured the Fe^2+^ accumulation levels in hippocampal tissue of mice from each group using an Fe^2+^ assay kit. Relative Fe^2+^ content of the PTZ group was significantly elevated compared with that in the CON and CON + PX-478 groups, whereas the PTZ + PX-478 group showed significantly reduced Fe^2+^ content relative to the PTZ group (**Figure 6A**). Subsequently, we assessed the expression of several specific ferroptosis molecular markers in hippocampal tissue using RT-qPCR and western blotting. At both transcriptional and translational levels, GPX4, prostaglandin-endoperoxide synthase 2 (PTGS2)/cyclooxygenase 2 (COX-2), ferritin heavy chain 1 (FTH-1), and transferrin (TRF) were significantly elevated in the PTZ group compared with those in the CON and CON + PX-478 groups, and were significantly decreased in the PTZ + PX-478 group compared with the PTZ group (**Figure 6B–D**). These findings suggest the presence of ferroptosis in hippocampal neurons following seizures, and that PX-478 treatment mitigated hippocampal neuronal ferroptosis.

### PX-478 decreases the expression of HIF-1α/HO-1 pathway in the hippocampus of epilepsy model mice

**Figure 7:**
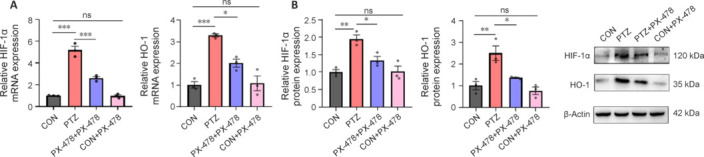
Effect of PX-478 on the expression of HIF-1α/HO-1 pathway in the hippocampus of epilepsy model mice. (A) RT-qPCR results for HIF-1α and HO-1 in the hippocampus. (B) Western blot results for HIF-1α and HO-1 in the hippocampus. The data were normalized to CON and presented as mean ± SEM (*n* = 3). *P < 0.05, **P < 0.01, ***P < 0.001 (one-way analysis of variance followed by Tukey’s honest significant difference test). CON: Control; HIF-1α: hypoxia-inducible factor 1α; HO-1: heme oxygenase-1; ns: not significant; PTZ: pentylenetetrazol; PX-478: a hypoxia-inducible factor-1α inhibitor; RT-qPCR: reverse transcription quantitative polymerase chain reaction.

Given the relationship between the HIF-1α/HO-1 pathway and ferroptosis (Wu et al., 2022), we investigated this pathway’s role in epilepsy pathology. We assessed the expression of the HIF-1α/HO-1 signaling pathway in hippocampal tissue using RT-qPCR and western blotting. Compared with the CON and CON + PX-478 groups, the PTZ group had significantly elevated mRNA and protein expression levels of HIF-1α and HO-1. In contrast, the PTZ + PX-478 group showed a significant reduction in HIF-1α and HO-1 expression compared with those in the PTZ group (**Figure 7A** and **B**).

## Discussion

Mitochondria are crucial for cellular energy production and maintaining redox balance (Hinton et al., 2024; You et al., 2024). In epilepsy, mitochondrial damage impairs mitochondrial function, reducing ATP production and disrupting calcium homeostasis. Mitochondrial dysfunction contributes to oxidative stress by increasing ROS production while simultaneously decreasing antioxidant defenses. Elevated ROS levels can lead to lipid peroxidation, protein oxidation, and DNA damage, creating a toxic environment that can trigger ferroptosis. The findings from the present study indicate that PX-478 alleviates epileptic seizures. In this study, compared with the PTZ group without PX-478 injection, the PTZ + PX-478 group showed significant improvements in four epilepsy behavioral observation indicators: complete seizure success rate, seizure latency period, latency from the last injection to seizure onset, and seizure duration. PX-478, as an orally active HIF-1α inhibitor, has primarily been studied for its anti-tumor activity and its mechanisms *in vivo*, particularly its impact on cellular function by inhibiting HIF-1α (Panahi Meymandi et al., 2024). However, there is currently no research on the application of PX-478 in epilepsy treatment or in alleviating epileptic seizure symptoms. Our study is the first to investigate PX-478 in epilepsy intervention and demonstrates its effectiveness in improving epileptic seizures, suggesting PX-478 as a potential antiepileptic drug and providing new insights for improving epilepsy treatment medications.

This study investigated the effects of PX-478 treatment on hippocampal neuronal damage in an epileptic mouse model. The findings showed that compared with the PTZ group, the PTZ + PX-478 group appeared to have reduced neuronal damage in the CA1 and CA3 regions of the hippocampus. However, some neuronal death and loss were still evident when compared with the CON group, confirming that epileptic seizures do lead to hippocampal neuronal death and loss, and that PX-478 injection mitigated this damage. PX-478, as a selective HIF-1α inhibitor, interferes with HIF-1α transcription and translation and inhibits HIF-1α deubiquitination (Lang et al., 2016). PX-478 has been shown to increase the radiosensitivity of prostate cancer cells and enhance the antitumor efficacy of gemcitabine in pancreatic ductal adenocarcinoma by inducing immunogenic cell death via HIF-1α inhibition (Zhao et al., 2015). When combined with dichloroacetate, PX-478 produces a synergistic therapeutic effect across various cancer cell lines, including colorectal, lung, breast, cervical, liver, and brain cancers. ROS generation and apoptosis play significant roles in this synergy, and PX-478 has been demonstrated to effectively inhibit cell proliferation (Parczyk et al., 2021). Furthermore, PX-478 has shown potential in improving neurological function. In a study by Yang et al. (2024) PX-478 treatment significantly improved spatial memory, learning, and social behavior in offspring rats exposed to fetal hypoxia, while also reducing anxiety-like behavior. HE staining showed that PX-478 treatment decreased the number of necrotic hippocampal neurons in these offspring. In addition, PX-478 treatment led to reduced HIF-1α protein levels in the hippocampus and lower serum vascular endothelial growth factor concentrations, alongside an increase in phosphatidylinositol-3-kinase inhibitor protein levels in hypoxia-exposed offspring rats. These studies collectively show that PX-478 exerts a variety of therapeutic effects through HIF-1α inhibition, including the effects observed in our study, where PX-478 injections improved hippocampal neuron damage in a mouse model of epilepsy.

To understand how PX-478 mitigates hippocampal neuronal damage in epileptic mice, this study investigated its effects on oxidative stress markers in the hippocampus. Lipid peroxidation signaling is considered a central mediator of ferroptosis. The level of lipid peroxidation can be assessed by detecting markers associated with oxidative damage (Tsuruta et al., 2024). ROS and MDA are critical biochemical indicators used to assess oxidative stress levels and lipid peroxidation extent in cells or tissues. Excessive ROS production is a pivotal component in the pathophysiology of progressive myoclonic epilepsy (Lehtinen et al., 2009), and increased ROS generation has been observed in the hippocampus and blood of patients with drug-resistant epilepsy and status epilepticus, indicating decreased antioxidant enzyme activity (Golyala and Kwan, 2017; Ray and Mukherjee, 2024). SOD, CAT, and GSH-Px are essential markers of antioxidant defense, playing crucial roles in metabolic and antioxidative processes in organisms (Wang et al., 2025). Multiple studies have reported significantly reduced SOD, CAT, and GPx activity in experimental epilepsy animals (Patel et al., 2001; Méndez-Armenta et al., 2014; Geronzi et al., 2018). Transgenic SOD^–/+^ mice exhibit increased susceptibility to epilepsy (Liang and Patel, 2004; Liang et al., 2012), and overexpression of mitochondrial SOD2 in mice shows protective effects against kainic acid-induced hippocampal neuron degeneration (Shin et al., 2008). The results of this study demonstrated that PX-478 injection alleviated hippocampal ROS and MDA accumulation and enhanced SOD, CAT, and GSH-Px levels in epileptic mice, suggesting that PX-478 enhances the antioxidant capacity of epileptic mice and exerts a beneficial effect on oxidative stress. Despite extensive evidence highlighting the role of oxidative damage in hippocampal neuronal injury following epilepsy, treatments targeting oxidative damage have shown limited clinical efficacy. Current clinical approaches primarily focus on antiepileptic therapy. The findings of this study underscore the potential of specific treatments that target oxidative damage in epilepsy, offering valuable insights for future therapeutic strategies aimed at mitigating oxidative stress in epilepsy.

Ferroptosis is closely associated with oxidative stress, and hippocampal neuronal ferroptosis is a key mechanism that triggers neuronal injury and epileptic seizures (Jiang et al., 2021). Morphologically and biologically distinct from other types of cell death, ferroptosis is characterized by smaller mitochondria with increased membrane density, reduced or absent cristae, mitochondrial outer membrane swelling, or rupture, while maintaining normal nuclear size without chromatin condensation (Shao et al., 2020). In this study, mice treated with PTZ + PX-478 exhibited longer and larger mitochondria than those in the PTZ group. Mitochondrial damage appeared to be reduced in the PTZ + PX-478 group compared with that in the PTZ group, suggesting that PX-478 injection mitigated ferroptosis, possibly by inhibiting ROS accumulation. ROS mainly originates from the mitochondrial respiratory chain, but excessive ROS directly attacks mitochondrial membrane lipids, proteins, and nucleic acids, leading to increased mitochondrial membrane permeability, decreased membrane potential, mitochondrial swelling, membrane rupture, and release of pro-apoptotic molecules into the cytoplasm, thereby triggering ferroptosis (Mao et al., 2019; Villalpando-Rodriguez and Gibson, 2021). Biochemically, ferroptosis involves iron accumulation and redox imbalance through multiple pathways, ultimately leading to lipid peroxidation (Wang et al., 2022). Intracellular antioxidants such as GSH are depleted, the cystine/glutamate antiporter system is downregulated, and GPX4 activity is reduced during ferroptosis. Consequently, lipid peroxides cannot be metabolized by GPX4-mediated reduction reactions, resulting in decreased cellular antioxidant capacity, accumulation of lipid ROS, and eventual ferroptosis (Xie et al., 2016; Chen et al., 2020; Cai and Yang, 2021). Additionally, Fe^2+^ plays a crucial role in ferroptosis by providing electrons to H_2_O_2_, triggering the Fenton reaction, which generates ROS and induces lipid peroxidation (Mittler, 2017). Under conditions of oxidative stress, Fe^3+^ stored mainly in ferritin undergoes the Haber–Weiss cycle, where superoxide O_2_^–^ reduces Fe^3+^ to Fe^2+^, exacerbating iron overload (Capelletti et al., 2020). Nrf2 plays a role in regulating Fe^2+^ levels in cells and has a protective effect against ferroptosis (Xie et al., 2016). The results of this study showed that PTZ + PX-478 treatment reduced Fe^2+^ content and PTGS2, FTH-1 and TRF expression compared with those in the PTZ group, but the levels were higher than those in the CON and CON + PX-478 groups. This result indicates that epilepsy leads to ferroptosis damage, and that PX-478 injection helps inhibit ferroptosis occurrence. These findings provide new theoretical insights and potential therapeutic targets for the diagnosis and treatment of epilepsy.

Our previous studies showed that the HIF-1α/HO-1 signaling pathway mediates hippocampal neuronal ferroptosis and participates in the onset and development of epilepsy (Liang et al., 2023a). The results of this study showed that HIF-1α and HO-1 expression in the PTZ + PX-478 group were lower than those in the PTZ group but higher than those in the CON and CON + PX-478 groups. This suggests that PX-478 injection inhibits the activation of the HIF-1α/HO-1 signaling pathway, which may be a mechanism through which it improves oxidative stress, reduces ferroptosis, alleviates hippocampal neuronal damage, and thereby improves epilepsy seizures. HIF-1α is a subunit of HIF-1, responsible for regulating cellular adaptive responses to hypoxic environments (Belaidi et al., 2016). HO-1, one of the key target genes of HIF-1α, is the sole pathway for cellular heme breakdown, metabolizing heme into biliverdin, carbon monoxide, and Fe^2+^ (which can induce ferritin increase) (Otterbein et al., 2003). Heme iron constitutes the major source of iron in the body (accounting for 67% of total body iron), and enhanced heme metabolism naturally leads to significant changes in body iron content, making HO-1 crucial in influencing these changes (Lawen and Lane, 2013). Elevated HO-1 can lead to ferrous iron overload, enhance the Fenton reaction, and convert normally less reactive O_2_^–^ and H_2_O_2_ into highly reactive OH, causing lipid peroxidation through excessive ROS attack on membranes, ultimately triggering ferroptosis (Chang et al., 2018; Hassannia et al., 2018). PX-478 inhibits HIF-1α through the following mechanisms: (1) by inhibiting the deubiquitination of HIF-1α, PX-478 promotes its ubiquitination, leading to the degradation of polyubiquitinated HIF-1α by the proteasome, thereby reducing cellular HIF-1α protein levels; and (2) by altering HIF-1α mRNA expression and translation, further lowering HIF-1α protein levels. HIF-1α can induce HO-1 expression. HO-1 plays a crucial role in cellular defense mechanisms, including oxidative stress and inflammation. GPX4 is an important antioxidant enzyme that plays a key role in protecting cells from ferroptosis caused by lipid peroxides. HIF-1α influences the levels of GPX4 by regulating the expression of downstream genes. HIF-1α may lead to a decrease in GPX4 expression, thereby weakening the cell’s ability to resist ferroptosis and increasing the risk of apoptosis and necrosis. FTH-1 and TRF are two key iron metabolism markers. Overall, the findings of the present study indicate that HIF-1α not only directly affects GPX4 expression but also alters the levels of iron metabolism-related markers.

The present study had several limitations. First, although animal models provide valuable insights, their relevance to human epilepsy pathology may be limited due to inherent species differences. Second, this study primarily focused on the acute effects of PX-478 treatment and did not extensively explore its long-term impacts and potential adverse outcomes. Future research should address these limitations through more comprehensive and translational approaches.

In summary, our findings suggest that PX-478 mitigates hippocampal neuronal ferroptosis by inhibiting HIF-1α, thereby improving PTZ-induced epileptic seizures.

## Data Availability

*No additional data are available*.
